# Pharmacovigilance in Israel – tools, processes, and actions

**DOI:** 10.1186/s13584-017-0154-3

**Published:** 2017-08-01

**Authors:** Eyal Schwartzberg, Matitiahu Berkovitch, Dorit Dil Nahlieli, Joseph Nathan, Einat Gorelik

**Affiliations:** 1 Pharmaceutical & Enforcement Divisions, Ministry of Health, 39 Yirmiyahu St., Jerusalem, Israel; 2 School of Pharmacy, Ben-Gurion University of the Negev, Beer sheeba, Israel; 3 Arnold and Marie Schwartz College of Pharmacy, Long Island University, Brooklyn, USA; 4 Clinical Pharmacology Unit, Assaf Harofeh Medical Center, Zerifin, an affiliate of Sackler School of Medicine, Tel-Aviv University, Tel-Aviv, Israel

**Keywords:** Pharmacovigilance, Adverse reactions reporting, Risk minimization, Signals, Israel

## Abstract

**Background:**

Due to the limited safety data available at the time that a new medication is first marketed, it is essential to continue the collection and monitoring of safety data about adverse drug reactions (ADRs) during the medication’s life cycle. This activity, known as pharmacovigilance (PV), is performed worldwide by the pharmaceutical industry as well as by regulatory agencies. In 2012, the Israeli Ministry of Health (MOH) established a Pharmacovigilance and Drug Information Department. The Department is tasked with identifying, monitoring, and initiating activities aimed at minimizing risks associated with medication utilization. To enable this, the MOH has devised procedures for PV and promoted extensive legislation in this area that require marketing authorization holders (MAHs) and medical institutions in Israel to report ADRs and new safety information to the MOH. A computerized database was created to support the reporting process. The objective of this article is to characterize the PV tools and activities implemented in Israel.

**Methods:**

Since September 2014, The Israeli Pharmacovigilance and Drug Information Department receives ICSRs at a central computerized database developed for this purpose. The data were analyzed by Department personnel and ICSRs were characterized according to their seriousness, source, categories of drugs involved, and the reporting format. Additionally, the Department reviewed signals detected from ADR reports and from other sources and assessed the resulting regulatory actions.

**Results:**

An analysis of the Individual Case Safety Reports (ICSRs) submitted to the MOH’s ADRs central database reveals that during the review period, a total of 16,409 ICSRs were received by the Department and 850 signals were identified, resulting in the following PV activities: inquiry and enhanced follow-up (430, 50.6%), prescriber’s and patient’s leaflets updates (204, 24%), recall of products/batches (6, 0.7%), alerts for health care professionals (63, 7.4%). Eighty five (10%) of the signals required a comprehensive investigation involving external specialist and 1 (0.1%) resulted in initiation of epidemiologic study. Additionally, in 2015 the Department incorporated comprehensive framework for risk minimization of marketed medicinal products, also known as risk management plans (RMPs).

**Conclusions:**

As practiced by other health authorities, the Israeli MOH effectively implemented various PV tools to ensure the safety of the Israeli health consumer.

## Background

At time of approval of a new medication, the information available about the product’s safety is based primarily on clinical trials. As such, the information is limited by many factors such as the trial’s sample size, duration of follow-up, as well as exclusion criteria. Generally, even the largest trials consist only of thousands of patients while real world utilization of a medication may involve millions of patients. Thus it is essential to routinely monitor and update medications’ safety profiles throughout their life cycle in order to ensure an optimal benefit to risk balance. Pharmacovigilance (PV) is defined by the World Health Organization (WHO) as the science and activities relating to the detection, assessment, understanding and prevention of adverse effects or any other drug-related problems [1]. Among the wide range of data sources used for PV activities, spontaneous adverse drug reaction (ADR) reports are most important for an early detection of risks associated with medication use.

### The international arena

In 1968, the WHO established its Programme for International Drug Monitoring in response to the notorious thalidomide catastrophe, which was detected in 1961. The program provides a forum for WHO member states to collaborate in the monitoring of drug safety, and notably, the identification and analysis of new adverse reaction signals from data submitted to the WHO global individual case safety report (ICSR) database by member countries [2]. WHO’s definition of signal is “Notice of an early concern or hypothesis about a possible medicines safety problem, with evidence and arguments to support it” [3]. The WHO promotes PV at an international level as well as on a country level. Globally, the WHO’s Collaborating Centre for International Drug Monitoring, located in Sweden, collects information from over 140 countries throughout Africa, the Americas, Asia, Australia, and Europe. The WHO’s global ICSRs database is called VigiBase. This database includes a web-based reporting and retrieval tool (VigiLyze) and an automated signal detection process. In April 2015, the WHO launched VigiAccess. VigiAccess is a new web application that will allow anyone to access information and encourage the reporting of adverse effects from medicinal products [1].

### Europe

Pharmacovigilance activities in Europe are overseen by the European Medicines Agency (EMA) as well as at states level. Each of the 28 member states in the EMA, operates a national PV system which collects and analyzes its findings locally and reports them centrally. The EMA also supports and coordinates the European PV system and consults on the safe and effective use of drugs. Pharmacovigilance information is gathered from various sources and stakeholders, including regulatory agencies, industry, healthcare professionals, as well as from consumers. At the EMA, the reports are assessed by the PV Risk Assessment Committee. This committee is responsible for evaluating and monitoring safety issues for human medicinal products and is composed of experts from regulatory authorities in member states, experts in medicine safety, healthcare professionals, and patient representatives [4].

The EMA’s reporting system for suspected cases of adverse reactions is the EudraVigilance. The EudraVigilance database includes reports of suspected adverse reactions to medicines from medical practice and from clinical trials. This system is not fully accessible by the general public and has different levels of access for different stakeholders [5].

### United States

The US Food and Drug Administration (FDA) Division of Pharmacovigilance assesses safety issues and detects signals for all marketed medicinal products in US. The FDA uses a variety of PV tools including post-marketing reports of adverse events, published scientific literature, and preclinical/clinical data to provide a comprehensive evaluation that leads to regulatory actions as well as safety communications concerning marketed products. The reports are maintained in the FDA Adverse Event Reporting System (FAERS). This database is designed to support the FDA’s post-marketing safety surveillance program for drug and therapeutic biologic products [6].

### Pharmacovigilance in Israel

Israel established a Pharmacovigilance and Drug Information Department within the Pharmaceutical Division of the MOH in 2012. Prior to this, reports on safety issues related to the use of medications were dealt with by various departments within the Pharmaceutical Division and not managed centrally. The Department was created in response to a series of multiple adverse events reported to the Ministry, highlighted by intense media and social media coverage; this was commonly referred to as the “levothyroxine (Eltroxin) event”. Upon establishment of the Department its manager underwent extensive training at the WHO’s center in Uppsala, Sweden. The Department also adopted the main principles of PV as practiced by the FDA and EMA.

The Pharmacovigilance and Drug Information Department is composed of pharmacists and clinical pharmacists who work in collaboration with the Clinical Pharmacology Department at Asaf Harofeh Medical Center in Israel. The department’s main functions include ADR and signal management, submission of safety evaluations of medicinal products to other departments within the MOH, implementation of risk minimization plans and communicating drug safety issues to healthcare professionals and to the public.

### Background: the levothyroxine event

In February 2011, the composition of the levothyroxine product, Eltroxin, which has been registered and marketed in Israel since 1981, was changed. Upon introduction of the new formulation in Israel, health care professionals began receiving reports of adverse events which were mostly related to hormonal imbalance (underactive thyroid, overactive thyroid) and a few involving allergic reactions. Since, the health care professionals were not aware of the change in the formulation, the adverse events were not attributed to the formulation of the medication. At the time, there was no requirement for medical institutions/health care providers to report suspected ADR to the MOH, therefore only some of the events were reported to the MOH. After additional investigation the ministry decided to take measures and instructed the MAH to raise awareness of health care providers and patients about the change and the need of close monitoring.

In the aftermath of the event, an investigation revealed that a similar change in formulation took place in Denmark in 2006 and in New Zealand in 2007. These changes also resulted in hundreds of reports of adverse events. Gaps in transferring this important PV information by the MAH to the Israeli Ministry of Health prevented preemptive measures that should have been taken by the Ministry.

In January 2012 an inquiry committee appointed by the Ministry’s Director General, published its report [7]. By then, 800 reports were received by the Pharmaceutical Division of the Ministry of Health. The committee emphasized that Israel had no requirement for medical institutions/health care providers to report ADRs to the Ministry of Health, as was the standard practice in other mature agencies worldwide, and noted the situation to be problematic. As such, it stressed the need to establish an infrastructure within the Pharmaceutical Division for collecting and analyzing adverse drug reactions reports and other safety data. The final report of the committee included recommendations to publish a procedure for reporting adverse events and safety information, to establish a PV and drug management department, to expand the collaboration with health authorities worldwide in the area of safety of medicinal products, to improve ways of communicating safety data to health care professionals and patients, and to increase the awareness among health care professionals of the importance of reporting adverse drug events. In light of the committee’s recommendations, the MOH established the Pharmacovigilance and Drug Information Department.

### Legislation and procedures for pharmacovigilance

Prior to establishing legislation on PV, the Ministry of Health followed procedures that were published in 2012 and were updated in February 2013. However these procedures were only aimed at the MAHs and excluded the obligation of health institutions to report the Ministry. In June 2013, the Israeli Parliament (“Knesset”) amended the Pharmacists Regulations (Medical Products) 1986, and anchored the obligations of all MAHs, health maintenance organizations (HMOs), and hospitals in Israel to report ADRs, or any new safety information relating to medications used in Israel to the MOH. This also included a requirement for the reporting to be done by a designated appointed person, also known as Qualified Person Responsible for Pharmacovigilance (QPPV), and for the organization to operate a drug safety monitoring system. As per the regulation, the QPPV has to be a pharmacist or a physician holding an Israeli license to practice, with practical experience of at least 2 years in his/her profession. This individual needs to be appointed by the organization and approved by the MOH. For situations involving medical gases, a practical engineer may also be appointed to this position.

Subsequently, In October 2013, the Pharmacovigilance and Drug Information Department published an updated version of the procedure for reporting adverse events and new safety information [8]. The updated version sets out PV requirements for the MAHs. It clarifies the work processes relating to ADR and other safety data reporting as well as the types of information which must be reported by the MAHs. In 2014, the Medical Affairs Administration of the MOH published a directive to medical directors of hospitals and HMOs regarding the importance of ADR reporting and guidance for establishing an ADR reporting system in their institutions.

In accordance with the aforementioned legislation, each of the 4 Israeli HMOs appointed a QPPV whose responsibility is to receive and collect reports from health care professionals within the institution and submit them to the MOH’s Pharmacovigilance and Drug Information Department. Clalit health services (“Clalit”), which serves more than 4 million citizens [9], is the largest HMO in Israel. Clalit is responsible for community care as well as secondary care and it operates a network of 14 hospitals in Israel. Clalit’s QPPV is also responsible for the collection and the reporting of ADRs from these hospitals. QPPVs at other HMOs are responsible for reports arising only at the primary care (community) settings. Currently, some of the hospitals have already appointed an in-house QPPV while, in others, the process of appointing a QPPV is ongoing.

## Methods

Since September 2014, The Israeli Pharmacovigilance and Drug Information Department receives ICSRs at a central computerized database developed for this purpose. The data were analyzed by Department personnel and ICSRs were characterized according to their seriousness, reporting source, categories of drugs involved, and the reporting format. Additionally, the Department reviewed signals detected from ADR reports and from other sources and assessed the resulting regulatory actions.

## Results

### ADR reporting system and ICSRs

Individual case safety reports are received by the Department from health care professionals, patients, and from the MAH. Between September 2014 and August 2016, 16,500 reports were entered into the PV central database. Of these reports, 94.3%were submitted by pharmaceutical companies (MAH and importers), 5.5% were submitted by health care professionals from medical institutions, and only 0.2% of the reports have been submitted by patients and the general public. In addition to the electronic reports received in the database, health care professionals also reported adverse events via mail or by phone and patients reported them as complaints or questions via the drug information call center or ADRs patients’ Forum, a web-based service offered by the PV department to the public (more than 130 per year). 40% of the ADR reports in the database, were defined as serious, 59% defined as non-serious, and for the other 1% seriousness was not specified.

Individual case safety reports can be submitted to the Pharmacovigilance and Drug Information Department by sending a standard International Conference on Harmonization (ICH) E2B format, an electronic format facilitated by the ICH for transmission of ADR reports. This method of transmission is currently used mainly by the industry (mostly MAH but also by some importers of unlicensed drugs which may be used in Israel due to drug shortages). Additionally the Department developed a designated electronic form allowing for an online reporting of ADRs by the general public, health care professionals as well as from QPPVs at various MAH/importers who do not have ICH E2B reporting system [10]. The form enables a structured method for reporting and filing an adverse event. The form is based on the WHO guidelines for effective and valid report. The minimum information for a valid report includes at least one patient identifier (initials, age, or gender), an identifiable reporter, at least one reaction, and at least one suspect drug. Individual case safety reports in both formats are received and displayed in the PV system described above.

Over the course of the assessment period, 50% of the reports were submitted via the online government form and 50% were submitted via E2B format. We performed an analysis of the ICSRs in our database according to Anatomical Therapeutic Chemical (ATC) classification – a classification of the suspected drug or active substance, according to the organ or system on which they act and their therapeutic, pharmacological and chemical properties [11]. The most frequently reported medication group was the immunosuppressant drugs (categorized by ATC -3). This is also the most frequent reported group in the WHO ICSRs database (VigiLyze).

**Fig. 1:**
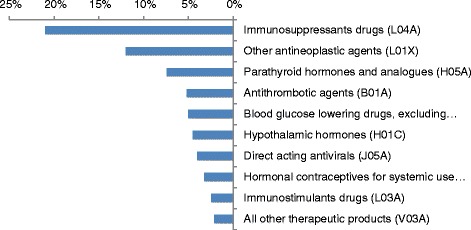
10 most common reported ATC-3 categories as percentage, of the total number of reports received to database from September 2014-August 2016

During the assessment period, the top 10 ATC-3 medication groups, which account for 68% of all ADRs reported to the department were immunosuppressant drugs (L04A), antineoplastic agents (L01X), parathyroid hormones and analogues (H05A), antithrombotic agents (B01A), blood glucose lowering drugs (excluding insulin; A10B), hypothalamic hormones (H01C), direct acting antivirals (J05A), hormonal contraceptives for systemic use (G03A), immunostimulant drugs (L03A), and all other therapeutic products (V03A). The latter medication group consists of antidotes, iron chelating agents, drugs for hypercalcemia, hyperkalemia and hyperphosphatemia, detoxifying agents, tissue adhesives, drugs for embolization, medical gases, nalfurafine, diazoxide, cobicistat and ethanol. Figure 1 presents the 10 most common ATC-3 medication groups as percentage, of the total number of reports received in database from September 2014 through August 2016.

### Signal management and regulatory actions

Signal detection is often based on a review of ICSRs. In the Pharmacovigilance and Drug Information Department, pharmacists and clinical pharmacists, specializing in PV, perform a case by case assessment to detect signals.

Additional sources for signals are reports of medication misuse and/or quality concerns arising from medical staff, industry-operated patient support programs, observational studies, clinical trials, ADRs patients’ forum, scientific literature, periodic safety reports/periodic benefit risk evaluation reports (a document submitted by the MAH to regulatory agencies), and safety communications from other health authorities (whose websites are monitored on daily basis). In order to confirm a signal, it is cross referenced with various sources of data. This process of signal detection, evaluation and prioritization takes into consideration factors such as frequency of the ADR, its severity, impact on public health, and the feasibility of counteract measures.

Additional factors taken into consideration include signals that involve serious, unexpected ADR, with reasonable causal association to the suspected drug, or a new aspect of a known association involving new product or new formulation. Moreover, the Department prioritizes signals derived from ADR reports that can indicate national safety issues such as a local quality concern and unusual frequency of a known ADR. Another important resource used by the Department to evaluate ADRs, signals, and other safety issues is an independent experts advisory committee appointed by the MOH for this purpose. Comparable committees exist at other regulatory agencies such as the FDA and EMA. The advisory committee consists of representatives from a variety of health disciplines, including clinical pharmacologists, pharmacists, and other specialists.

In addition, Israel has signed agreements with several leading medicines agencies, such as Swissmedic and the FDA. These memorandums of understanding include PV data sharing. Overall, when needed, the Department communicates safety dilemmas with other regulatory agencies, mainly the EMA, FDA and Swissmedic. Following an internal assessment process and additional consultation with other regulatory agencies (if needed), the Department concludes whether the evidence is sufficient to support a safety concern to the public and determines what steps should be taken to manage and minimize this potential risk.

Since 2014 until the end of 2016, 850 signals have been identified from all sources. Following evaluations that took into account the impact on public health and the feasibility of counteract measures, it was determined that 430 signals required inquiry or follow-up and 420 of the signals required additional regulatory actions. Forty percent of the signals originated from ADR reports.

**Fig. 2:**
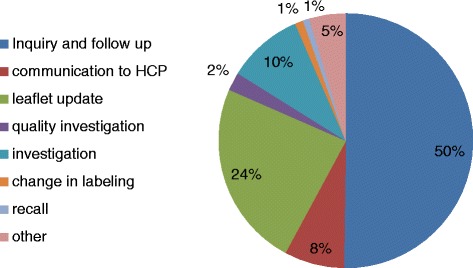
Distribution of PV activities initiated as a response to signals (*n* = 850)

Among the PV activities initiated following the signal detection were: initiating leaflets and label updates, informing health care providers and the public regarding safety concerns, and in special cases –withdrawing a drug from the market, recalls or inventory holds of specific products or batches. Figure 2 represents the distribution of PV activities initiated in response to signals (*n* = 850).

In addition to the described activities, as of 2015 the Pharmacovigilance and Drug Information Department takes proactive risk management process to minimize potential risks associated with the use of medications by implementing risk minimization plans (RMPs) with the MAHs. RMPs are key regulatory tool for characterization, assessment, mitigation, and minimization of risks associated with use of a medicinal products. A procedure that sets the guidelines for submitting RMPs to the Pharmaceutical Division was published in March 2016. Since 2015 to date, 30 RMPs were implemented in Israel.

### Examples of PV activities based on signal detection from ADR reports and/or other sources

#### Increased frequency of labeled ADRs

In 2014 the Department received 11 reports in a period of 10 days regarding urticaria, which presented as intense itchy skin rash, and developed during or minutes to hours after the infusion of a specific batch of an immunoglobulin product. In the prescribing information, urticaria is listed as an uncommon adverse reaction of immunoglobulin products. An investigation was initiated which revealed no quality issues that may have caused the above ADR. Nevertheless, due to the unexplained unusual frequency of these serious drug events, the Department decided to hold the marketing of this batch. Following this hold, there were no additional reports.

#### Rare and severe ADRs

Three spontaneous reports of death associated with intramuscular administration of benzathine benzylpenicillin (penicillin G benzathine) had been received by the Department during a period of 9 months (2014–2015). The 3 cases involved elderly female patients aged 91, 77 and 70 years with underlying cardiac disease but in stable clinical condition. The patients were treated for erysipelas, with monthly intramuscular injections of the benzathine benzylpenicillin diluted with lidocaine. In each of the cases, shortly after the intramuscular injection of Benzathine benzylpenicillin, the patient lost consciousness with respiratory and cardiac arrest. After a thorough investigation among the 4 HMOs, 3 more cases of severe adverse reactions to benzathine benzylpenicillin during occurring within the last 3 years were identified; one of which was fatal. The benzathine benzylpenicillin was imported from different manufacturers and was from different batches. Chemical analysis of the suspected products revealed no quality issues. In all cases the benzathine benzylpenicillin was diluted with a small amount of lidocaine 1% in order to minimize the pain associated with the injection but the amount of lidocaine injected could not explain this adverse reaction. In each case, the drug was administered for at least several months prior to the event and therefore an evaluation performed by the PV expert advisory committee concluded that anaphylaxis was not a reasonable explanation for the events. It should be noted that the product leaflet contains strict administration instructions for the drug. Specifically, it states that the drug may only be administered by deep intramuscular injection and that intravenous administration of penicillin G benzathine has been associated with cardiopulmonary arrest and death. After considering the available data, it was determined that a possible explanation of the events might have been cardiopulmonary arrest due to embolism after inadvertent intravascular injection. Considering the benefit to risk evaluation, the MOH decided that the risk outweighs the benefit when administering this agent for most indications. In 2015, the MOH issued a communication regarding the restriction of using benzathine benzylpenicillin for a limited number of infections such as syphilis, where other alternative therapies are not suitable [12].

#### External sources of information received from other regulatory agencies

In June of 2016, the FDA issued a communication that emphasized the existing warning about acute kidney injury with the sodium glucose transporter 2 (SGLT2) inhibitors canagliflozin and dapagliflozin, which are used for treatment of diabetes mellitus [13]. A question has been raised about the third member of the SGLT2 inhibitors, empagliflozin. The FDA communication was based on recent reports regarding the aforementioned drugs. Empaglifozin was not included in the FDA review or in a similar review conducted by Health Canada [14], most likely due to the fact that it was new in the market. The Pharmacovigilance and Drug Information Department communicated this dilemma to its colleagues in Swissmedic and the EMA. Neither agency planned to issue a similar communication regarding a similar risk with empagliflozin. Following a review of the safety data from clinical trials, post-marketing data, and the medical literature, the Department and the advisory committee concluded that the information regarding volume depletion, renal adverse reactions and the necessity to monitor renal function that is currently mentioned in the Israeli product leaflets for all 3 agents is sufficient and that there was no need to issue further communication similar to the FDA and Health Canada to the health care providers in Israel.

#### Drug-Lab interactions

Fulvestrant is indicated for the treatment of breast cancer. Due to a structural similarity between fulvestrant and estradiol, fulvestrant may interfere with antibody-based estradiol assays and may result in falsely increased levels of estradiol. Following a report on an unnecessary surgical procedure performed in a patient who was treated with fulvestrant which resulted in falsely elevated estradiol levels, the MOH ordered the company to issue a communication letter to health care professionals regarding this cross reactivity. The letter emphasized the importance of informing the laboratory performing the estradiol immunoassay about the treatment with fulvestrant and viewing the results of the estradiol levels in correlation with the clinical status of the patient. The product leaflets will be updated accordingly.

#### Emerging crisis due to pharmaceutical compounding

In July 2014, the Department received 3 reports of infection with Pantoea in children treated with total parenteral nutrition (TPN) prepared by the largest compounding pharmacy in Israel. After a special request that was made to the medical institutions for similar reports regarding pantoea infections in TPN users, additional 8 validated cases were received. The MOH with the compounding pharmacy collected the suspected batches and initiated a comprehensive investigation including examination of manufacturing process, equipment, and the sterility of each component. Medical institutions were instructed to switch patients to alternative products and to report adverse events related to TPN treatment. This crisis was managed by multidisciplinary team from various professions, expertise, and institutions. Following corrective activities, the compounding pharmacy resumed its routine manufacturing. Since this crisis, as a preventive measure, the MOH instructed the preparation centers to check sterility of samples from each prepared batch.

#### Initiating and deploying a national risk minimization plan

The MOH receives numerous reports of hemorrhagic events involving new oral anticoagulants (NOACs). Many of these events are serious and some are partially preventable. Because of these reports and in light of safety communications issued by some regulatory agencies, in 2014 the Department decided to initiate a risk minimization plan in collaboration with MAHs of NOAC products. The plan included prescriber guides, patient cards, and education plans for health care professionals. These educational materials were designed to emphasize the most important safety information, contraindications, main risk factors for bleeding, recommendations for conversion from vitamin K antagonists, recommendations for dosing adjustments, and more. The plans were implemented successfully in Israel in 2015.

#### Increased reporting due to previous high profile ADR event

Following the Eltroxin incident, the MOH supported the registration of additional levothyroxine products in order to provide therapeutic alternatives to patients with hypothyroidism in Israel. Two new products were registered in 2013. Since then, a few clusters of ADR reports for levothyroxine products were received by the Department. One of these clusters was received in 2014 and involved the alteration of the external packaging information of a specific product. As a response to that cluster, the Department initiated an inquiry, involving the MAH and the Institute for Standardization and Control of Pharmaceuticals of the MOH. The inquiry did not reveal any quality issues, changes in the formulation, or deviation from specifications. According to the MAH there has been no a change in the manufacturing site or the manufacturing process. It seems that the change in the information on the packaging triggered the ADR reports. Prior to its registration, the product was marketed in Israel with a US label and after its registration, it was marketed with a Canadian label. After completing the investigation, a communication was issued to health care providers informing them about the inquiry findings and the differences in labeling that were due to different labeling requirement of the 2 regulatory authorities.

In 2015 a second cluster of ADR reports including chest pain, gastrointestinal complaints, headache, and general “bad feeling” was received concerning a specific batch of levothyroxine. The Department initiated a new inquiry. Samples from new packages and used packages from different batches were examined by the Institute for Standardization and Control of Pharmaceuticals of the MOH. This inquiry also did not reveal any quality problem, changes in the formulation, or deviations from specifications. Following a clinical consultation with the advisory committee, no causal association was found between the medication and the complaints. No further action was required.

In 2016, a MAH of levothyroxine announced a change in the external packaging for marketing reasons. As a preventive measure, a communication about the change was issued to health care professionals and patients. The communication stressed the fact that no other change was made. Nevertheless, since this communication another cluster of ADR reports was received. The Department continues to monitor and evaluate these reports and will take actions as needed.

## Discussion

The Pharmacovigilance and Drug Information Department was established with a mindset for optimal use of national resources for effective identification, communication, and minimization of risk. This is manifested in the form of initial and ongoing robust education and training program for the Department’s personnel, establishing a legal framework for reporting, managing and communicating safety and risk data, benchmarking its activities to international standards, and creating partnerships and close collaborations with leading medicines agencies and the academy. These activities were supported by an extensive information technology infrastructure as well as by creating a network of experts in the field, advising on ongoing and emerging risks.

Unfortunately, only a minority of ADRs are reported to regulatory agencies. ADR reporting behavior varies between countries but, in general, it is believed that approximately 5–10% of all adverse reactions are actually reported [15, 16]. One of the Department’s goals is to raise awareness of the importance of ADR recognition and reporting in order to increase reporting rates across the sectors. For this purpose, the Department’s personnel lecture to health care professionals and industry and communicate safety information when needed. Appointment of QPPVs within the pharmaceutical companies and medical institutions is also aimed at increasing awareness of the importance of ADR reporting among the medical staff.

As noted earlier, the Department developed an electronic form allowing for an online reporting of ADRs. The form is available on the MOH website. In the near future, the Department plans to implement a new version of the online reporting form with a simplified adapted version for patients and health care professional in order to facilitate reporting.

In order to optimize the process of signal detection from high volume data, various statistical methods have been developed for routine monitoring of big data for signal detections. The MOH devotes resources to update the PV system and develop online forms to enable such quantitative signal detection. For example, implementation of the MedRA medical coding dictionary in the new version of the electronic reporting form will allow a quick and efficient identification, evaluation and analysis of ADR reports. However, it is important to point out that currently such decision support capacity is not aimed at replacing clinical judgment of PV experts.

Future attempts will be aimed at gathering safety information from non-conventional sources of data such as social media, apps, and real world evidence derived from patients and patients groups. While this may be challenging, it will assist in bridging the gap in real time reporting and enable the MOH to capture the broad picture while utilizing big data for the purpose of PV.

## Conclusions

The Israeli Pharmacovigilance and Drug Information Department is tasked with identifying, monitoring, and minimizing adverse outcomes associated with the use of medical products. The PV approaches described in this article have been implemented successfully in Israel. The main strategies for risk minimization are publication of safety alerts, updates of prescriber’s and patient leaflets and development and approval of additional risk minimization plans. Efforts are ongoing to update the surveillance system and to improve the online forms to enable more effective ADR reporting and signal detection. The Department will continue its collaboration with international regulatory agencies to promote the health of the Israeli public.

## Abbreviations

ADRs: Adverse drug reactions
ATC: Anatomical Therapeutic Chemical
EMA: European Medicines Agency
FAERS: FDA Adverse Event Reporting System
FDA: Food and Drug Administration
HMO: Health maintenance organization
ICH: International Conference on Harmonization
ICSRs: Individual case safety reports
MAH: Marketing authorization holder
MOH: Ministry of Health
NOAC: New oral anticoagulant
PRAC: Pharmacovigilance Risk Assessment Committee
PV: Pharmacovigilance
QPPV: Qualified Person Responsible for Pharmacovigilance
RMP: Risk management plan
SGLT2: Sodium glucose transporter 2
TPN: Total parenteral nutrition
WHO: World Health Organization
